# Protection of Dietary Polyphenols against Oral Cancer

**DOI:** 10.3390/nu5062173

**Published:** 2013-06-14

**Authors:** Yijian Ding, Hua Yao, Yanan Yao, Leonard Yenwong Fai, Zhuo Zhang

**Affiliations:** 1 Department of Stomatology, First Affiliated Hospital, Zhejiang University, Hangzhou 310003, China; E-Mails: hzdyj@yahoo.com.cn (Y.D.); asdfgh257@gmail.com (Y.Y.); 2 Graduate Center for Toxicology, University of Kentucky, Lexington, KY 40536, USA; E-Mail: leonardfai@uky.edu

**Keywords:** dietary polyphenols, tea polyphenols, (−)-epigallocatechin-3-gallate, oral cancer, cancer prevention, cancer therapy, cell death, cell growth, cell migration and invasion

## Abstract

Oral cancer represents a health burden worldwide with approximate 275,000 new cases diagnosed annually. Its poor prognosis is due to local tumor invasion and frequent lymph node metastasis. Better understanding and development of novel treatments and chemo-preventive approaches for the preventive and therapeutic intervention of this type of cancer are necessary. Recent development of dietary polyphenols as cancer preventives and therapeutic agents is of great interest due to their antioxidant and anti-carcinogenic activities. Polyphenols may inhibit carcinogenesis in the stage of initiation, promotion, or progression. In particular, dietary polyphenols decrease incidence of carcinomas and exert protection against oral cancer by induction of cell death and inhibition of tumor growth, invasion, and metastasis. In this review, we discuss current progress of dietary polyphenols against oral cancers *in vitro*, *in vivo*, and at population levels.

## 1. Introduction

### 1.1. Oral Cancer

Oral cancer is a serious and growing issue in many countries [1,2]. Oral and pharyngeal cancer, grouped together, is the sixth most common cancer in the world. The annual estimated incidence of oral cancer is about 275,000; two-thirds of these cases occurred in developing countries. Both incidence and mortality rate are higher in men than women. The variety of incidence and mortality rate among countries may due to distinct risk profiles, and availability and accessibility of health services. In the areas with high incidence rate such as Sri Lanka, India, Pakistan, and Bangladesh, oral cancer is the most common cancer in men, and may contribute up to 25% of all new cases of cancer [3]. Most oral malignancies are squamous cell carcinomas and the risk of developing oral cancer has been shown to increase with age [3]. Tobacco use including smokeless tobacco and excessive alcohol consumption are estimated to account for about 90% of oral cancers [3]. The 58th World Health Assembly Resolution on Cancer Prevention and Control in year of 2005 urged Member States to develop and reinforce national cancer control program, prioritizing preventable tumors and risk factor intervention.

### 1.2. Polyphenols

Polyphenols represent the largest group of phytochemicals found in plants, particularly in fruits, seeds, and leaves. These compounds exhibit health benefits through complementing and adding to the functions of antioxidant vitamins and enzymes as defense against oxidative stress caused by excess reactive oxygen species (ROS). Dietary polyphenols have been paid attention due to their benefits on human health. High intake of fruits, vegetables, and whole grains, which are rich in polyphenols, has been linked to lowered risks of many diseases including cancer, cardiovascular disease, chronic inflammation, and neurodegenerative diseases [4]. In particular, the property of dietary polyphenols against tumorigenesis is attractive to the researchers and health workers. The action of polyphenols has been investigated in various cancers including lung, skin, oral cavity, ovarian, esophagus, stomach, liver, pancreas, endometrial, thyroid, testicular bladder, small intestine, colon, urinary tract, and prostate [5,6]. It has been demonstrated that polyphenols exhibit anti-cancer property by interfering with molecular events involved in initiation, promotion, and progression stages [7].

Chemoprevention is a promising approach for controlling transformation of premalignant or potentially malignant lesions to invasive cancer. Polyphenols has been used as a chemopreventive agent to modulate oral carcinogenesis process. In this review, we provide an overview of protective effects of dietary polyphenols against oral cancers.

## 2. Classification and Resources of Dietary Polyphenols

Dietary polyphenols constitute one of the most numerous and widely distributed groups of natural products in the plant kingdom. Fruits, vegetables, whole grains, and beverages such as tea, chocolate, and wine are rich sources of polyphenols. More than 8000 phenolic structures are currently known, and majority of plants carry at least some of polyphenols. Although polyphenols are chemically characterized as compounds with phenolic structural features, this group of natural products is highly diverse.

### 2.1. Phenolic Acids

Phenolic acids are abundant in food and may account for about one third of phenolic compounds in our diet. They can be further divided into two main types: hydroxybenzoic acid and hydroxycinnamic acid derivatives based on C1–C6 and C3–C6 backbones. Caffeic acid and ferulic acid are main hydroxycinnamic acid derivatives. Caffeic acid, which can be found in many fruits such as apple, plum, tomato, and grape, occurs mainly as an ester with quinic acid called chlorogenic acid (5-caffeoylquinic acid). Chlorogenic acid is a key substrate for enzyme oxidation. Ferulic acid, which is linked through ester bonds to hemicellulose of the cell wall, is found in grains and seeds. Curcumin, another hydroxycinnamic acid derivative, is used for food preservation with yellow pigment in turmeric and mustard [8].

### 2.2. Flavonoids

Flavonoids contain the C6–C3–C6 general structural backbone in which the two C6 units (Rings A and B) are of phenolic nature. These compounds constitute a very diverse group of secondary plant metabolites and they are divided into sub-groups including flavonols, flavones, flavanones, flavanonols, flavanols, isoflavonoids, anthocyanidins, and proanthocyanidins [9,10,11]. Flavones and their 3-hydroxy derivative flavonols are the largest subgroup among all polyphenols. Quercetin is common flavonol present in many fruits, vegetables, and beverages [12]. Flavanones and their 3-hydroxy derivatives flavanonols have a large number of substituted derivatives [13]. Flavanols or flavan-3-ols are commonly called catechins, which are abundant in tea, red wine, and chocolate. (+)-Catechin and (−)-epicatechin are two isomers found in food plants, particularly in skins of grape, apple, and blueberry [14]. Anthocyanidins are principal components of red, blue, and purple pigments of flower petals, fruits, and vegetables, and varieties of grains, such as black rice. 90% of anthocyanins are based on cyanidin, delphinidin, and pelargonidin and their methylated derivatives [15].

### 2.3. Resveratrol, Lignans and Ellagic Acid

Resveratrol is a plant-derived polyphenolic phytoalexin produced by enzyme stilbene synthase. It is present in grape, red wine and peanut [16]. Lignans exist in the bound forms in flax, sesame, and many grains. Ellagic acid, a dimer of gallic acid, can be found in free forms in different plants [17].

## 3. Dietary Intake of Phenolic Compounds

Because polyphenols represent a wide variety of diverse structures from different subclasses, it is difficult to estimate total polyphenol content. It had been reported that main polyphenol dietary sources are fruits and beverages (fruit juice, wine, tea, coffee, chocolate, and beer) and, to a lesser extent, vegetables, dry legumes, and cereals. The total intake is ~1 g/day [1]. The mean intake of polyphenols was 863 ± 415 mg/day based on 48 hour dietary recalls in 2007 [18]. Phenolic acids comprised dominant group of polyphenols (75% of total intake) followed by proanthocyanidins (14%), anthocyanidins, and other flavonoids (10%). Coffees and cereals are key contributors to the total polyphenol intake especially to phenolic acids. Berries and their products are main sources for anthocyanidins, ellagitannins, and proanthocyanidins, while fruits are sources for flavonols, flavones, and flavanones [18]. Pierre *et al.* [19] generated a database of polyphenol content of fruits and vegetables and then evaluated polyphenol intake through fruit and vegetable consumption in France. Based on fruit consumption data, apples and strawberries are main sources of polyphenols in French diet, whereas potatoes, lettuces, and onions are the most important vegetable sources. Total polyphenol intake from fruits is about three times higher than from vegetables. The calculation of polyphenol intake showed that apples and potatoes provide ~47% of the total polyphenol intake from fruit and vegetables in French diet [19]. Overall, the consumption of dietary polyphenols can vary significantly among individual due to different dietary habits.

## 4. Bioavailability of Phenolic Compounds

The bioavailability of phenolics is low and the values of urinary excretion of intake range from 0.3% for anthocyanins to 43% for isoflavones [20]. The absorption of food phenolics is determined primarily by chemical structure, which depends on degree of glycosylation and acylation, basic structure (*i.e.*, benzene or flavone derivatives), conjugation with other phenolics, molecular size, polymerization, and solubility [21]. Maximum concentration in plasma rarely exceeds 1 mM after the consumption of 10–100 mg of a single phenolic compound [22]. It has been reported that a micromolar concentration of quercetin, catechins, isoflavones in blood was reached after a meal comprising onions, red wine, and soy, respectively [1,23,24].

## 5. Dietary Polyphenol and Oral Cancer Prevention

Many studies have been carried out to investigate inhibitory effect of plant polyphenols on tumorigenesis. In these studies, polyphenols-mediated oncogenes, tumor suppressor genes, cell cycle, apoptosis, angiogenesis, and related signal transduction pathways are emphasized. In this review, we only discuss studies related to oral cancer.

### 5.1. In Vitro Studies

A variety of *in vitro* studies demonstrated multiple outcomes as mechanisms of polyphenols. Major *in vitro* studies that show the targets/outcomes of chemoprevention by polyphenols are summarized in Table 1 and discussed below.

#### 5.1.1. EGCG and other Green Tea Polyphenols

Tea is listed to the second only to water in worldwide popularity as a beverage. Green tea was shown to be associated with many health benefits including prevention of cancer and heart disease for decades [2]. Major constitutions of green tea are (−)-epigallocatechin gallate (EGCG), (−)-gallocatechin (EGC), (−)-epicatechin gallae (ECG), (−)-catechin gallate (CG), (−)-epicatechin (EC), and (+)-catechin (C) [25]. Numerous *in vitro* studies have shown capability of green tea polyphenols especially EGCG in reduction of cell growth, induction of apoptosis, and inhibition of angiogenesis in oral cancer cell lines [26]. EGCG acts as a pro-oxidant and tumor cells are more vulnerable to oxidative forces than normal cells. Avi *et al.* [27] reported that EGCG effectively inhibited cancer cell growth in cells derived from oral dysplastic leukoplakia and squamous cell carcinoma. Jeffrey *et al.* [28] have also reported that the anti-proliferative effect of green tea polyphenols and EGCG, was more sensitive in oral cancer cell lines (CAL27, HSC-2, and HSG1) than normal fibroblasts (GN56 and HGF-1). EGCG treatment caused generation of hydrogen peroxide (H_2_O_2_), one of major ROS. The results from assessment of cytotoxicity of a green tea polyphenol CG in cell lines derived from human oral cavity indicated that CG selectively induced cell death in favor of cancer cells [29]. Further study indicated that cytotoxicity of CG in cancer cells was due to its capability of inducing H_2_O_2_ generation.

**Table 1:** Summary of *in vitro* studies of dietary polyphenols against oral cancer.

Compound	Cell line(s)	Treatment dose/duration	Targets/Outcome (Reference)
BTEs	SCC-4	10–40 μg/mL, 24 h	Reducing MMP-2 and uPA [30]
Cranberry polyphenols	KB and CAL27	200 µg/mL, 48 h	Inhibiting oral cancer cells [31]
CG and other TPs	S-G, CAL27, and HSG	25–200 µM, up to 72 h	Inducing cell death and inhibiting proliferation [29]
DMF	SCC-9	0.1–100 µM, up to 48 h	Inhibiting CYP1B1/1A1 function [32]
ECG and other TPs	HSC-2 and HGF-2	50–500 µM, up to 72 h	Inhibiting cell proliferation and inducing apoptosis [33]
EGCG	CAL-27	25–100 μM, up to 48 h	Suppressing p-EGFR and MMP-2 [34]
EGCG	SCC-9	5–20 µM, up to 24 h	Reducing expressions of MMP-2, MMP-9, uPA, p-FAK, Src, snail-1, and vimentin [35]
EGCG	Tu177, Tu212, Tu686 , and 686LN	30 µM, up to 72 h	Inducing cell cycle arrest and apoptosis via p53 [36]
EGCG	HSC3, HSC4, SCC9, SCC25	5–50 µM, up to 5 days	Enhancing RECK expression, inhibiting MMP-2 and MMP-9 expression [37]
EGCG	OC2	5–60 µM, 24 h	Inhibiting cell invasion and migration [38]
EGCG	OSC2, G6, S2, and S5	50 µM, up to 72 h	Regulating p21WAF1, p57 modulating epithelial cell differentiation, or apoptosis [39,40]
EGCG	OECM-1	1–20 µM, 24 h	Inhibiting APP expression [26]
EGCG	NS-SV-AC, NSSV, OSC-2, and OSC-4	12.5–50 µM, 24 h	Protecting cells from chemical or irradiation-induced damage [41]
EGCG and curcumin	MSK Leuk1	50 µM, 5 days	Cell growth [27]
EGCG, ECG, EGC, resveratrol, and quercetin	SCC-25	50–200 µM, up to 72 h	Cell growth [42,43]
GTE and EGCG	CAL-27, SCC-25, and KB	50-200 µM, up to 72 h	Inhibiting cell growth via EGFR and Notch signaling [44]
Polyphenon-E and Polyphenon-B	CAL-27	25–400 µg/mL, 24 h	Inducting ROS generation and Bcl-2/Bax-mediated apoptosis [45]
GTPs and EGCG	OSC2	50–200 µM, up to 72 h	Inducing apoptosis, inhibiting cell growth [46,47,48]
Methoxylated flavones	SCC-9	25 µM, 24 h	Inhibiting CYP1B1 mRNA expression [49]
MT	SCC-61 and OSCC-3	0.05–1000 µg/mL, 48 h	Inhibiting oral cancer proliferation [50]
150 of natural and synthetic polyphenols	HSC-2, HSG	Various, up to 48 h	Cytotoxic activity [51,52]
Quercetin	SCC-9 cells	0–200 µM, up to 72 h	Inducing cell death [53]
Quercetin, resveratrol, and ellagic acid	Oral tissue	25 µM, 24 h	Inhibiting BaP-DNA binding and oxidization [54]
Sasa senanensis Rehder leaves extracts	HSC-2	0.22% and 0.18%, 24 h	Protecting cells from ultraviolet -induced injury [55]
TF-2A and TF-2B	HSC-2 and CAL27	100–500 µM, up to 24 h	Prooxidant action [56,57]
TPs	Tca8113	25–200 µM, up to 72 h	Inhibiting cell proliferation and hTERT expression [58,59]
Tea extracts and EGCG	CAL27, HSC-2, HSG, S-G, GN56, and HGF-1	0–200 µg/mL, up to 72 h	Oxidative stress-induced cell death [28]
TPs and EGCG	KB-A-1	0.2 µg/mL, 24 h	Enhancing intracellular concentration of DOX and modulating MDR [60]

Elattar *et al.* [42] evaluated the effect of three major tea constituents, EGCG, ECG, and EGC on the cell growth and DNA synthesis of human oral squamous carcinoma cell line SCC-25. These three compounds caused dose-dependent inhibition in both cell growth and DNA synthesis [42]. A study by Muneyuki *et al.* [61,62] has also demonstrated that treatment with EGCG induced cell cycle arrest at G1 phase due to decrease of cyclin D1 expression, increases of p21Cip1 and p27Kip1 expression, and reduction of hyper-phosphorylated form of pRB. Liu *et al.* [44] have found that green tea extract and EGCG inhibited cell growth in three squamous cell lines (CAL-27, SCC-25, and KB) via S and G2/M phase cell cycle arrest. Results from Pathway Array assessment of 107 proteins indicated that major signaling pathways affected by GTE and EGCG were EGFR and Notch, which in turn affected cell cycle related networks [44].

Epithelial to mesenchymal transition (EMT) is critical for the progression, invasion, and metastasis of epithelial tumorigenesis. Molecular evidences associated with the anti-metastatic effect of green tea polyphenols and EGCG have been established. EGCG treatment in oral squamous SCC-9 cells blocked cell invasion via a reduced expression of matrix metalloproteinase-2 (MMP-2) and urokinase type plasminogen activator (uPA) [35]. EGCG exerted an inhibitory effect on cell migration, motility spread, and adhesion. EGCG inhibited phospho-focal adhesion kinase (p-FAK), p-Src, snail-1, and vimentin, indicating the anti-EMT effect of EGCG in oral squamous cell carcinoma [35]. RECK is a tumor suppressor gene that negatively regulates MMPs and inhibits tumor invasion, angiogenesis, and metastasis. Kato *et al.* [37] have reported that treatment of oral cancer cells with EGCG partially reversed the hypermethylation status of the RECK gene and significantly enhanced the expression level of RECK mRNA, leading to reduced MMP-2 and MMP-9 expressions.

In addition to its capacity against tumorigenesis, EGCG also synergistically cooperated with other pharmaceutical inhibitors, such as gefitinib, an EGFR inhibitor. Combined treatment of gefitinib and EGCG synergistically inhibited invasion and migration of CAL-27 cells [34]. EGCG sensitized CAL-27 cells to gefitinib-suppressed phosphorylation of EGFR [34]. Another study has also showed that simultaneous treatment with EGCG and erlotinib, an inhibitor of EGFR-tyrosine kinase, strongly induced cell cycle arrest and apoptosis via p53-dependent induction of p21, p27, and Bim, and p53-dependent inhibition of NF-κB and its antiapoptotic target, Bcl-2 in squamous cell carcinoma of the head and neck cells (SCCHN) [36].

Resistance of malignant tumors to chemotherapeutic agents is a major cause of treatment failure in patients with cancers. Wei *et al.* [60] demonstrated that tea polyphenols (TPs) and EGCG were modulators of multidrug resistance (MDR) that could influence activity of anti-neoplastic drug, doxorubicin (DOX). DOX, the most effective broad spectrum antitumor antibiotics, its application in clinic may be limited due to MDR. In fact, TPs and EGCG enhanced the intracellular concentration of DOX by 4.4 and 2.2 times, respectively. Overexpression of amyloid precursor protein (APP) is a biomarker to monitor OSCC prognosis. Shun-Yao *et al.* [26] have demonstrated that EGCG might diminish oral carcinogenesis by down-regulating APP in OECM-1 oral squamous carcinoma cells. Babich *et al.* [58] and Yao *et al.* [59] have shown that TPs disabled telomerase activity and thereby terminated unlimited cancer cell proliferation in cancerous Tca8113 cells, giving additional insight into the inhibitory potential of tea ingredients in the treatment of resistant tumors.

#### 5.1.2. Black Tea Polyphenols

The typical composition of black tea beverage contains 8% of catechins, 10% of flavonol glycosides, 12% of theaflavins, and 70% of thearubigens [63]. Among these components, theaflavins are generally considered to be most effective against carcinogenesis. Schuck *et al.* [56] have shown that treatments of theaflavin-3 and 30-digallate (TF-3) inhibited more cell growth in HSC-2 cancer cells than normal GN46 fibroblasts. TF-3 could generate ROS, leading to apoptotic cell death in HSC-2 cells, but not in GN46 fibroblasts. TF-3 may function as a prooxidant to induce oxidative stress, thereby accounting for their cytotoxicity to cancerous cells [56].

The responses of human gingival fibroblasts and carcinoma cells derived from the tongue to theaflavin-3-gallate (TF-2A) and theaflavin-3′-gallate (TF-2B), two polyphenols in black tea, have been compared [57]. It was reported that TF-2A and TF-2B acted as pro-oxidants to induce cell apoptosis in carcinoma cells, but not in normal fibroblasts [57]. ROS played a key role and that Bcl-2/Bax was involved in mitochondrial mediated apoptosis by dissipating mitochondrial transmembrane potential (ΔΨm) and activating caspase-3 [46]. Chang *et al.* [30] have demonstrated that inhibition of black tea polyphenol extracts (BTE) on cell invasion in human tongue squamous carcinoma SCC-4 cells was through reduced activities of MMP-2 and u-PA, up-regulated E-cadherin, and down-regulated snail and vimentin.

#### 5.1.3. Other Dietary Polyphenols

Besides green and black tea polyphenols, many other dietary polyphenols are associated with oral cancer-preventive activities. A total of 150 chemically defined natural and synthetic polyphenols (flavonoids, dibenzoylmethanes, dihydrostilbenes, dihydrophenanthrenes, and 3-phenylchromen-4-ones) were investigated for cytotoxic activity against normal, tumor, and HIV-infected cells [51]. Similar to tea polyphenols, these polyphenols selectively caused more cell death in cancer cells than that in normal cells [51].

Resveratrol and quercetin are polyphenols that have been detected in significant amounts in green vegetables, citrus fruits, and red grape wines. Resveratrol induced significant dose-dependent inhibition of DNA synthesis and related cell growth while quercetin exerted a biphasic effect: stimulation at 1 μM and 10 μM, and minimal inhibition at 100 mM in cell growth and DNA synthesis [43]. Haghiac *et al.* [53] reported that quercetin induced necrosis at treatment time of 24 h and 48 h, whereas at 72 hours cells underwent apoptosis, correlating with caspase-3 activation. Study on cell cycle distribution showed that quercetin induced S-phase arrest with an inhibition of thymidylate synthase, a key enzyme of S phase. Walle *et al.* [54] have tested quercetin, resveratrol, and ellagic acid in a bioengineered construct of the multilayered stratified human oral epithelial tissue. The results showed that flavonoids, 5,7-dimethoxyflavone (5,7-DMF) and 3′,4′-dimethoxyflavone (3′,4′-DMF) were able to inhibit benzo[a]pyrene (BaP)-induced CYP1A1 and 1B1 activities [32]. The results from screening of 19 methoxylated falvones in human squamouse cell carcinoma SCC-9 cell showed that 4 out of 19 compounds including 3′,4′-DMF, 5,7,4′-trimethoxyflavone, 7,3′-dimethoxyflavone, and 7,4′-dimethoxyflavone were found to be potent inhibitors of CYP1B1 mRNA expression [49]. Curcumin and quercetin, unmethylated polyphenols, were also potent inhibitors [49].

### 5.2. Animal Studies

At present, there are a limited number of animal studies showing the usefulness of dietary polyphenols on oral cancer chemoprevention by modulating cell proliferation, cell survival, tumor infiltration, angiogenesis, and apoptosis. Important work on this direction are discussed below and summarized in Table 2.

7,12-dimethylbenz (a) anthracene (DMBA)-induced hamster buccal pouch (HBP) carcinogenesis model has been extensively employed to investigate the chemopreventive effectiveness of dietary agents. Ning *et al.* [64] has shown that tea preparations could inhibit DMBA-induced oral carcinogenesis in hamsters effectively due to their capacities against DNA damage and suppression of cell proliferation. Chandra *et al.* [65] and Mohan *et al.* [66] observed that inhibition of HBP carcinomas by polyphenon-E and -B was associated with a significant decrease in phase I enzymes, modulation of lipid peroxidation and enhanced antioxidant and phase II enzyme activities. Administration of polyphenon-E and -B to DMBA-treated hamsters significantly decreased lipid peroxidation and enhanced GSH level, GSH/GSSG ratio, and activities of GSH-dependent enzymes [66,67]. Polyphenon-B was more effective in inhibiting HBP carcinogenesis than polyphenon-E by enhancing the antioxidant status [67]. Dietary supplementation of polyphenon B could exert protection against DMBA-induced genotoxicity and oxidative stress by augmenting bone marrow antioxidant defense mechanisms [68].

Moreover, the inhibitory effects of polyphenon-B on HBP carcinogenesis has been associated to a decrease in cell proliferation and an enhancement in apoptosis, as evidenced by down-regulations of PCNA, NF-κB, p53, and Bcl-2 and upregulation of Bax, Fas, and caspase 3 [69]. Letchoumy *et al.* [70] found that administration of polyphenon-B and BTF-35 significantly decreased tumorigenesis induced by DMBA by reduced expressions of p21, cyclin D1, GST-P, NF-κB, cytokeratins, VEGF, and Bcl-2, and increased expressions of Bax, cytochrome C, caspase-3, caspase-9, and PARP.

Other *in vivo* studies have been undertaken in addition to DMBA-induced HBP carcinogenesis. Srinivasan *et al.* [71] reported that green tea polyphenols (GTPs) administration to rats significantly reduced both number and volume of oral squamous cell carcinomas induced by 4-nitroquinoline 1-oxide (4-NQO) compared to 4-NQO treatment only. Inhibition of Phase I enzymes (cytochrome b5, cytochrome P450, and cytochrome b5 reductase) and induction of Phase II enzymes ((glutathione-S-transferase and UDPglucuronyl transferase) by GTPs could be attributed to their protective efficacy against tumorigenesis [71]. GTPs reduced oxidant production and increased levels of GSH, PSH, and total thiols [72]. Other studies have shown that GTPs administration altered the expression of glycoconjugates (hexose, hexosamine, sialicacid, fucose and mucoprotein) and immunological markers (circulating immune complex and mast cell density), resulting in regression of oral cancer [73]. In a hamster model of *N*-methyl-*N*-benzylnitrosamine (MBN)-induced oral carcinogenesis, the incidence of buccal pouch carcinomas was significantly reduced when administrated with EGCG [26].

**Table 2:** Summary of *in vivo* studies of dietary polyphenols against oral cancer.

Compound	Animal studies	Treatment dose, route, and duration	Target/Outcome (Reference)
EGCG	Immuno-deficient nude mice	10–20 mg/kg/day, oral gavage, 45 days	Inhibition of tumor growth and cell invasion [35]
EGCG	MBN-treated HBP carcinomas	0.2%, drinking water, 9 weeks	Decreasing the incidence of carcinomas and APP expression [26]
EGCG and EGC	Wistar strain rats	200 mg/kg/day, oral gavage, 13 weeks	Inhibiting Phase I enzymes to deactivate carcinogen, inducing Phase II enzymes to detoxify 4-NQO [71]
(−)-Gossypol	Athymic nude mice	5 and 15 mg/kg/day, intraperitoneal, 91 days	Inhibiting tumor growth [74]
GTPs	DMBA-induced oral carcinogenesis in golden Syrian hamsters	0.5%–1.5%, drinking water, 15 weeks	Inhibiting oral carcinogenesis, protecting from DNA damage and suppression of cell proliferation [64]
GTPs	Wistar strain rats	200 mg/kg/day, oral gavage, 30 days	Reducing oxidant production and enhancing cellular thiol status to mitigate oral cancer and attenuating MC activation [72,73]
Polyphenon-B	DMBA-induced HBP carcinogenesis	0.05% and 0.2%, diet, 14 weeks	Decreasing cell proliferation and enhancing apoptosis by downregulating PCNA, NF-κB, p53 and Bcl-2 and upregulating Bax, Fas and caspase 3 expression [69]
Polyphenon-B and BTF-35	DMBA-induced HBP carcinogenesis	0.05% and 0.2%, diet, 14 weeks	Inhibiting oxidative DNA damage and modulating xenobiotic-metabolizing enzymes [75]
Polyphenon-E and Polyphenon-B	DMBA-induced HBP carcinogenesis	0.05%, diet, 18 weeks	Inhibiting HBP carcinogenesis and modulating carcinogen-metabolizing enzymes and the redox status [65,67,68]

### 5.3. Clinical Studies and Epidemiologic Studies

Although many laboratory studies have demonstrated that dietary polyphenols exhibit anti-mutagenic and anti-carcinogenic effects, intervention trial is the most important approach to elucidate protective effects of dietary polyphenols. A number of clinical studies and epidemiologic studies have been summarized in Table 3.

Ning *et al.* [76] performed the first clinical trial in 1999 to provide direct evidence of the protective effect of tea polyphenols against human oral cancer. A double-blind intervention trial was conducted in 64 patients of both sexes, mostly smokers with oral mucosa leukoplakia (a pre-neoplastic lesion) using a mixed green tea product compared to the placebo (using starch and coloring agents with only glycerin) for six months. Results showed that oral lesions were significantly reduced in 38% of patients treated with green tea mixture compared with 10% of patients in the placebo group. The incidence of micronucleated exfoliated oral mucosal cells was significantly lowered in the green tea mixture-treated group (5.4/1000 cells) compared to that in control placebo group (11.3/1000 cells). The overall results provided a unique opportunity for the investigation of mechanisms of oral cancer prevention by dietary polyphenols in humans. Schwartz *et al.* [77] performed a pilot intervention study using human oral cells in three heavy smokers (A10 cigarettes/day) and three nonsmokers (without smoking history) in order to evaluate the molecular and cellular effects of administrating green tea total extracts (2000–2500 mg/day). The results showed that green tea administration decreased smoking-induced DNA damage and inhibited cell growth. This study verified the role of green tea in the prevention of oral cancer in smokers by inducing cancer cell growth arrest and apoptosis, a mechanism similar to that observed in cultured cells and animal models.

Tsao *et al.* [78] performed a phase II randomized, placebo controlled clinical trial of green tea extract (GTE) (500, 750, or 1000 mg/m^2^) in patients with high risk oral premalignant lesions (OPL) for 12 weeks. The OPL clinical response rate was higher in all GTE arms versus placebo but did not reach statistical significance. However, the two higher-dose GTE arms (750 and 1000 mg/m^2^) had higher response, suggesting dose-response effect of GTE on oral premalignant lesions. Baseline stromal VEGF levels correlated with clinical but not histologic response. Other biomarkers including epithelial VEGF, p53, Ki-67, cyclin D1, and p16 promoter methylation) were not associated with the outcome. Results from this study suggested that GTE might suppress OPLs, in part through reducing angiogenic stimulus (stromal VEGF). Another clinic trial study indicated that green tea exhibited protective effects against oxidative DNA damage induced by cigarette smoking, leading to delayed lesion progress in patients with oral mucosa leukoplakia, demonstrating its protective effects from oral cancer [79].

In addition to the preventive effects of green tea, the chemopreventive capacity of other dietary polyphenols has also been investigated. Halder *et al.* [80] have explored the possible benefits of black tea administered to 82 patients with oral leukoplakia. The first 15 patients enrolled in this study showed a significant decrease in the micronuclei frequency and chromosomal aberrations, which correlated with the clinical improvement [80].

**Table 3:** Summary of epidemiologic and clinical studies of dietary polyphenols against oral cancer.

Compound	Human studies	Administration dose, route, duration, and cases	Target/Outcome (Reference)
TPs	Study in patients with cigarette smoking and oral mucosa leukoplakia	3 g/day. Both oral and topical administration. 6 months. 59 patients.	Protection against oxidative damage and DNA damage caused by cigarette smoking, blocked lesion progress in patients with oral mucosa leukoplakia [79]
GTE	Phase II randomized, placebo controlled clinical trial in high risk oral premalignant lesions (OPLs)	500 mg/m^2^, 750 mg/m^2^, and 1 g/m^2^. Oral administration. 3 months. 11 controls and 30 patients with oral premalignant lesions.	Suppress OPLs, in part through reducing angiogenic stimulus (stromal VEGF) [78]
Isoflavones, anthocyanidins, flavan-3-ols, flavanones, flavones, and flavonols	Case-control study about oral and pharyngeal cancer risk	24.8 µg isoflavones, 21.9 mg anthocyanidins, 65.4 mg flavan-3-ols, 38.8 mg flavanones, 0.5 mg flavones, 22.5 mg flavonols, and 149.2 mg total flavonoids. Diet. 13 years. 2081 controls and 805 patients.	Decreasing the probability to develop oral and pharyngeal cancers by 50% [81]
Green tea	Prospective, large-scale cohort study	1–5 cups/day. Drinking water. 10.3 years. 65,184 subjects.	Reduced the hazard ratios (HRs) of oral cancer in women [82]
Green tea	Pilot intervention study with heavy smokers	400–500 mg/cup, 5 cups/day. Drinking water. 4 weeks. 3 control, 3 patients.	Inducing cell growth arrest and apoptosis [77]
Black tea	Clinical Trial in patients with oral leukoplakia	3 teaspoon s/day. Drinking water. 1 year. 82 patients with precancerous lesion.	Decreasing micronuclei frequency and chromosomal aberrations [80]
Mixed green tea	Double-blind intervention trial performed in patients with oral mucosa leukoplakia	3 g/day. Oral administration. 6 months. 30 control, 29 oral mucosa leukoplakia.	Reducing oral leukoplakia in size, inhibiting cell micronucleated exfoliation [76]

Furthermore, the relation between dietary polyphenols and the risk of oral cancer was also investigated by some epidemiologic studies. An Italian case-control study has been conducted to examine the relationship between flavonoid intake to oral and pharyngeal cancer risk from 1992 to 2005 [81]. This study included 805 cases confirmed with oral and pharyngeal cancer, and 2081 hospital controls were admitted for non-neoplastic conditions. Results have shown that a high dietary flavonoid intake decreased the probability of developing oral and pharyngeal cancers by about 50% [81]. Ide *et al.* [82] conducted a prospective nationwide, large-scale cohort study in Japan including 20,550 men and 29,671 women aged 40–79 years without any history of oral cancer. After an average of 10.3 years follow-up period, tea consumption group showed chemo-preventive effects in women according to the reduction of hazard ratios of oral cancer when increasing green tea dietary administration per day. However, no such trends were identified in men [82].

## 6. Conclusions

This review summarizes the preventive and possible therapeutic properties of dietary polyphenols against oral cancer *in vitro*, *in vivo*, and in clinical studies. Oral cancer is one of the most important medical problems. Chemopreventive strategies represent a promising approach to reduce the incidence and mortality of this disease. Most studies have demonstrated that dietary polyphenols could regulate numerous molecular pathways involved in cancer promotion and progression, suggesting chemopreventive and therapeutic capacity of dietary polyphenols against oral cancers. Among all polyphenols investigated, tea polyphenols have been shown to have obvious anti-carcinogenic effects. Limited investigations of other dietary polyphenols have been conducted in spite of their diversity and rich sources. Scientific evidences on other dietary polyphenols should be demonstrated to clarify the potentials of these molecules on prevention of oral cancer. Compared to a large amount of *in vitro* and animal studies, only a few clinical trials have been performed. Additional randomized clinical trials and cohort studies should be conducted to obtain direct evidence of dietary polyphenols against oral cancers.

## Abbreviations

4-NQO: 4-nitroquinoline 1-oxide
APP: Amyloid precursor protein
BaP: Benzo[*a*]pyrene
BTE: Black tea polyphenol extracts
C: (+)-Catechin
CDDP: cis-Platinum(II) diammine dichloride
CG: (−)-Catechin gallate
DMBA: 7,12-Dimethylbenz (a) anthracene
EC: (−)-Epicatechin
ECG: (−)-Epicatechin gallae
EGC: (−)-Gallocatechin
EGCG: Epigallocatechin gallate
EGFR: Epidermal growth factor receptor
EMT: Epithelial to mesenchymal transition
ERK: Extracellular regulated kinase
FAK: Focal adhesion kinase
GTE: Green tea extract
GTPs: Green tea polyphenols
HBP: Hamster buccal pouch
MBN: *N*-methyl-*N*-benzylnitrosamine
MDR: Multidrug resistance
MMP-2: Matrix metalloproteinase-2
MMP-9: Matrix metalloproteinase-9
MMPs: Matrix metalloproteinases
OPL: Oral premalignant lesions
ROS: Reactive oxygen species
Stat 3: Signal transducer and activator of transcription 3
SCCHN: Squamous cell carcinoma of the head and neck
TF-2A: Theaflavin-3-gallate
TF-2B: Theaflavin-3′-gallate
TF-3: Theaflavin-3 and 30-digallate
TPs: Tea polyphenols
uPA: Urokinase-type plasminogen activator

## References

[1] Siegel R., Naishadham D., Jemal A. (2012). Cancer statistics, 2012. CA Cancer J. Clin..

[2] Petersen P.E. (2005). Strengthening the prevention of oral cancer: The who perspective. Community Dent. Oral Epidemiol..

[3] Warnakulasuriya S. (2009). Global epidemiology of oral and oropharyngeal cancer. Oral Oncol..

[4] Scalbert A., Williamson G. (2000). Dietary intake and bioavailability of polyphenols. J. Nutr..

[5] Yang C.S., Maliakal P., Meng X. (2002). Inhibition of carcinogenesis by tea. Annu. Rev. Pharmacol. Toxicol..

[6] Arts I.C., Hollman P.C. (2005). Polyphenols and disease risk in epidemiologic studies. Am. J. Clin. Nutr..

[7] Lambert L.D., Hong J., Yang G.Y., Liao J., Yang C.S. (2005). Inhibition of carcinogenesis by polyphenols: Evidence from laboratory inverstigation. Am. J. Clin. Nutr..

[8] Khan N., Adhami V.M., Mukhtar H. (2010). Apoptosis by dietary agents for prevention and treatment of prostate cancer. Endocr. Relat. Cancer.

[9] D’Archivio M., Filesi C., di Benedetto R., Gargiulo R., Giovannini C., Masella R. (2007). Polyphenols, dietary sources and bioavailability. Ann. Ist. Super Sanita.

[10] Chun O.K., Chung S.J., Song W.O. (2007). Estimated dietary flavonoid intake and major food sources of us adults. J. Nutr..

[11] Erdman J.W., Balentine D., Arab L., Beecher G., Dwyer J.T., Folts J., Harnly J., Hollman P., Keen C.L., Mazza G. (2007). Flavonoids and heart health: Proceedings of the ilsi north america flavonoids workshop, May 31–June 1, 2005, Washington, DC. J. Nutr..

[12] Cimino S., Sortino G., Favilla V., Castelli T., Madonia M., Sansalone S., Russo G.I., Morgia G. (2012). Polyphenols: Key issues involved in chemoprevention of prostate cancer. Oxid. Med. Cell Longev..

[13] Kawaii S., Tomono Y., Katase E., Ogawa K., Yano M. (1999). Quantitation of flavonoid constituents in citrus fruits. J. Agric. Food Chem..

[14] Tsao R., Yang R., Young J.C., Zhu H. (2003). Polyphenolic profiles in eight apple cultivars using high-performance liquid chromatography (HPLC). J. Agric. Food Chem..

[15] Andersen O.M., Jordheim M., Andersen O.M., Markham K.R. (2006). The Anthocyanins. Flavonoids: Chemistry, Biochemistry, and Applications.

[16] Athar M., Back J.H., Tang X., Kim K.H., Kopelovich L., Bickers D.R., Kim A.L. (2007). Resveratrol: A review of preclinical studies for human cancer prevention. Toxicol. Appl. Pharmacol..

[17] Tsao R. (2010). Chemistry and biochemistry of dietary polyphenols. Nutrients.

[18] Ovaskainen M.L., Torronen R., Koponen J.M., Sinkko H., Hellstrom J., Reinivuo H., Mattila P. (2008). Dietary intake and major food sources of polyphenols in finnish adults. J. Nutr..

[19] Brat P., George S., Bellamy A., Du Chaffaut L., Scalbert A., Mennen L., Arnault N., Amiot M.J. (2006). Daily polyphenol intake in france from fruit and vegetables. J. Nutr..

[20] Manach C., Mazur A., Scalbert A. (2005). Polyphenols and prevention of cardiovascular diseases. Curr. Opin. Lipid..

[21] Karakaya S. (2004). Biovailability of phenolic compounds. Crit. Rev. Food Sci. Nutr..

[22] Landete J.M. (2012). Updated knowledge about polyphenols: Functions, bioavailability, metabolism, and health. Food Sci. Nutr..

[23] Rowland I., Faughnan M., Hoey L., Wahala K., Williamson G., Cassidy A. (2003). Bioavailability of phyto-oestrogens. Br. J. Nutr..

[24] Williamson G., Manach C. (2005). Bioavailability and bioefficacy of polyphenols in humans. II. Review of 93 intervention studies. Am. J. Clin. Nutr..

[25] Yang C.S., Wang Z.Y. (1993). Tea and cancer. J. Natl. Cancer Inst..

[26] Ko S.-Y., Chang K.-W., Lin S.-C., Hsu H.-C., Liu T.-Y. (2007). The repressive effect of green tea ingredients on amyloid precursor protein (APP) expression in oral carcinoma cells *in vitro* and *in vivo*. Cancer Lett..

[27] Khafif A., Schantz S.P., Chou T.C., Edelstein D., Sacks P.G. (1998). Quantitation of chemopreventive synergism between (−)-epigallocatechin-3-gallate and curcumin in normal, premalignant and malignant human oral epithelial cells. Carcinogenesis.

[28] Weisburg J.H., Weissman D.B., Sedaghat T., Babich H. (2004). *In vitro* cytotoxicity of epigallocatechin gallate and tea extracts to cancerous and normal cells from the human oral cavity. Basic Clin. Pharmacol. Toxicol..

[29] Babich H., Zuckerbraun H.L., Weinerman S.M. (2007). *In vitro* cytotoxicity of (−)-catechin gallate, a minor polyphenol in green tea. Toxicol. Lett..

[30] Chang Y.C., Chen P.N., Chu S.C., Lin C.Y., Kuo W.H., Hsieh Y.S. (2012). Black tea polyphenols reverse epithelial-to-mesenchymal transition and suppress cancer invasion and proteases in human oral cancer cells. J. Agric. Food Chem..

[31] Seeram N.P., Adams L.S., Hardy M.L., Heber D. (2004). Total cranberry extract *versus* its phytochemical constituents: Antiproliferative and synergistic effects against human tumor cell lines. J. Agric. Food Chem..

[32] Wen X., Walle T. (2005). Preferential induction of cyp1b1 by benzo[*α*]pyrene in human oral epithelial cells: Impact on DNA adduct formation and prevention by polyphenols. Carcinogenesis.

[33] Babich H., Krupka M.E., Nissim H.A., Zuckerbraun H.L. (2005). Differential *in vitro* cytotoxicity of (−)-epicatechin gallate (ECG) to cancer and normal cells from the human oral cavity. Toxicol. In Vitro.

[34] Chang C.M., Chang P.Y., Tu M.G., Lu C.C., Kuo S.C., Amagaya S., Lee C.Y., Jao H.Y., Chen M.Y., Yang J.S. (2012). Epigallocatechin gallate sensitizes cal-27 human oral squamous cell carcinoma cells to the anti-metastatic effects of gefitinib (Iressa) via synergistic suppression of epidermal growth factor receptor and matrix metalloproteinase-2. Oncol. Rep..

[35] Chen P.N., Chu S.C., Kuo W.H., Chou M.Y., Lin J.K., Hsieh Y.S. (2011). Epigallocatechin-3 gallate inhibits invasion, epithelial-mesenchymal transition, and tumor growth in oral cancer cells. J. Agric. Food Chem..

[36] Amin A.R., Khuri F.R., Chen Z.G., Shin D.M. (2009). Synergistic growth inhibition of squamous cell carcinoma of the head and neck by erlotinib and epigallocatechin-3-gallate: The role of p53-dependent inhibition of nuclear factor-kappab. Cancer Prev. Res. (Phila.).

[37] Kato K., Long N.K., Makita H., Toida M., Yamashita T., Hatakeyama D., Hara A., Mori H., Shibata T. (2008). Effects of green tea polyphenol on methylation status of reck gene and cancer cell invasion in oral squamous cell carcinoma cells. Br. J. Cancer.

[38] Ho Y.C., Yang S.F., Peng C.Y., Chou M.Y., Chang Y.C. (2007). Epigallocatechin-3-gallate inhibits the invasion of human oral cancer cells and decreases the productions of matrix metalloproteinases and urokinase-plasminogen activator. J. Oral Pathol. Med..

[39] Yamamoto T., Digumarthi H., Aranbayeva Z., Wataha J., Lewis J., Messer R., Qin H., Dickinson D., Osaki T., Schuster G.S., Hsu S. (2007). EGCG-targeted p57/KIP2 reduces tumorigenicity of oral carcinoma cells: Role of c-Jun *N*-terminal kinase. Toxicol. Appl. Pharmacol..

[40] Hsu S., Farrey K., Wataha J., Lewis J., Borke J., Singh B., Qin H., Lapp C., Lapp D., Nguyen T., Schuster G. (2005). Role of p21WAF1 in green tea polyphenol-induced growth arrest and apoptosis of oral carcinoma cells. Anticancer Res..

[41] Yamamoto T., Staples J., Wataha J., Lewis J., Lockwood P., Schoenlein P., Rao S., Osaki T., Dickinson D., Kamatani T., Schuster G., Hsu S. (2004). Protective effects of egcg on salivary gland cells treated with gamma-radiation or cis-platinum(II)diammine dichloride. Anticancer Res..

[42] Elattar T.M., Virji A.S. (2000). Effect of tea polyphenols on growth of oral squamous carcinoma cells *in vitro*. Anticancer Res..

[43] ElAttar T.M., Virji A.S. (1999). Modulating effect of resveratrol and quercetin on oral cancer cell growth and proliferation. Anticancer Drugs.

[44] Liu X., Zhang D.Y., Zhang W., Zhao X., Yuan C., Ye F. (2011). The effect of green tea extract and egcg on the signaling network in squamous cell carcinoma. Nutr. Cancer.

[45] Mohan K.V., Gunasekaran P., Varalakshmi E., Hara Y., Nagini S. (2007). *In vitro* evaluation of the anticancer effect of lactoferrin and tea polyphenol combination on oral carcinoma cells. Cell Biol. Int..

[46] Hsu S., Lewis J., Singh B., Schoenlein P., Osaki T., Athar M., Porter A.G., Schuster G. (2003). Green tea polyphenol targets the mitochondria in tumor cells inducing caspase 3-dependent apoptosis. Anticancer Res..

[47] Hsu S.D., Singh B.B., Lewis J.B., Borke J.L., Dickinson D.P., Drake L., Caughman G.B., Schuster G.S. (2002). Chemoprevention of oral cancer by green tea. Gen. Dent..

[48] Hsu S., Lewis J.B., Borke J.L., Singh B., Dickinson D.P., Caughman G.B., Athar M., Drake L., Aiken A.C., Huynh C.T., Das B.R., Osaki T., Schuster G.S. (2001). Chemopreventive effects of green tea polyphenols correlate with reversible induction of p57 expression. Anticancer Res..

[49] Walle T., Walle U.K. (2007). Novel methoxylated flavone inhibitors of cytochrome P450 1B1 in SCC-9 human oral cancer cells. J. Pharm. Pharmacol..

[50] Gonzalez de Mejia E., Song Y.S., Ramirez-Mares M.V., Kobayashi H. (2005). Effect of yerba mate (Ilex paraguariensis) tea on topoisomerase inhibition and oral carcinoma cell proliferation. J. Agric. Food Chem..

[51] Fukai T., Sakagami H., Toguchi M., Takayama F., Iwakura I., Atsumi T., Ueha T., Nakashima H., Nomura T. (2000). Cytotoxic activity of low molecular weight polyphenols against human oral tumor cell lines. Anticancer Res..

[52] Ito H., Kobayashi E., Takamatsu Y., Li S.H., Hatano T., Sakagami H., Kusama K., Satoh K., Sugita D., Shimura S., Itoh Y., Yoshida T. (2000). Polyphenols from eriobotrya japonica and their cytotoxicity against human oral tumor cell lines. Chem. Pharm. Bull. (Tokyo).

[53] Haghiac M., Walle T. (2005). Quercetin induces necrosis and apoptosis in SCC-9 oral cancer cells. Nutr. Cancer.

[54] Walle T., Walle U.K., Sedmera D., Klausner M. (2006). Benzo[*a*]pyrene-induced oral carcinogenesis and chemoprevention: Studies in bioengineered human tissue. Drug Metab. Dispos..

[55] Matsuta T., Sakagami H., Kitajima M., Oizumi H., Oizumi T. (2011). Anti-UV activity of alkaline extract of the leaves of *Sasa senanensis* rehder. In Vivo.

[56] Schuck A.G., Ausubel M.B., Zuckerbraun H.L., Babich H. (2008). Theaflavin-3,3′-digallate, a component of black tea: An inducer of oxidative stress and apoptosis. Toxicol. In Vitro.

[57] Babich H., Gottesman R.T., Liebling E.J., Schuck A.G. (2008). Theaflavin-3-gallate and theaflavin-3′-gallate, polyphenols in black tea with prooxidant properties. Basic Clin. Pharmacol. Toxicol..

[58] Yao H., Wang H.M., Wu Q.L., Fan J. (2005). Effects of tea polyphenols on cell proliferation and hTERT of human tca8113 cell lines. Zhonghua Kou Qiang Yi Xue Za Zhi.

[59] Yao H., Li J.H., Wu Q.L, Fan J., Zhi C. (2006). Effects of tea polyphenols on telomerase activity of a tongue cancer cell line: A preliminary study. Int. J. Oral Maxillofac. Surg..

[60] Wei D., Mei Y., Liu J. (2003). Quantification of doxorubicin and validation of reversal effect of tea polyphenols on multidrug resistance in human carcinoma cells. Biotechnol. Lett..

[61] Masuda M., Suzui M., Lim J.T.E., Deguchi A., Soh J.-W., Weinstein I.B. (2002). Epigallocatechin-3-gallate decreases vegf production in head and neck and breast carcinoma cells by inhibiting EGFR-related pathways of signal transduction. J. Exp. Ther. Oncol..

[62] Masuda M., Suzui M., Weinstein I.B. (2001). Effects of epigallocatechin-3-gallate on growth, epidermal growth factor receptor signaling pathways, gene expression, and chemosensitivity in human head and neck squamous cell carcinoma cell lines. Clin. Cancer Res..

[63] Wiseman S., Mulder T., Rietveld A. (2001). Tea flavonoids: Bioavailability *in vivo* and effects on cell signaling pathways *in vitro*. Antioxid. Redox Signal..

[64] Li N., Han C., Chen J. (1999). Tea preparations protect against dmba-induced oral carcinogenesis in hamsters. Nutr. Cancer.

[65] Chandra Mohan K.V., Hara Y., Abraham S.K., Nagini S. (2005). Comparative evaluation of the chemopreventive efficacy of green and black tea polyphenols in the hamster buccal pouch carcinogenesis model. Clin. Biochem..

[66] Mohan K.V., Letchoumy P.V., Hara Y., Nagini S. (2008). Combination chemoprevention of hamster buccal pouch carcinogenesis by bovine milk lactoferrin and black tea polyphenols. Cancer Investig..

[67] Chandra Mohan K.V., Subapriya R., Hara Y., Nagini S. (2006). Enhancement of erythrocyte antioxidants by green and black tea polyphenols during 7,12-dimethylbenz[*a*]anthracene-induced hamster buccal pouch carcinogenesis. J. Med. Food.

[68] Letchoumy P.V., Chandra Mohan K.V., Kumaraguruparan R., Hara Y., Nagini S. (2006). Black tea polyphenols protect against 7,12-dimethylbenz[*a*]anthracene-induced hamster buccal pouch carcinogenesis. Oncol. Res..

[69] Chandra Mohan K.V., Devaraj H., Prathiba D., Hara Y., Nagini S. (2006). Antiproliferative and apoptosis inducing effect of lactoferrin and black tea polyphenol combination on hamster buccal pouch carcinogenesis. Biochim. Biophys. Acta.

[70] Letchoumy P.V., Mohan K.V., Prathiba D., Hara Y., Nagini S. (2007). Comparative evaluation of antiproliferative, antiangiogenic and apoptosis inducing potential of black tea polyphenols in the hamster buccal pouch carcinogenesis model. J. Carcinog..

[71] Srinivasan P., Suchalatha S., Babu P.V., Devi R.S., Narayan S., Sabitha K.E., Shyamala Devi C.S. (2008). Chemopreventive and therapeutic modulation of green tea polyphenols on drug metabolizing enzymes in 4-nitroquinoline 1-oxide induced oral cancer. Chem. Biol. Interact..

[72] Srinivasan P., Sabitha K.E., Shyamaladevi C.S. (2004). Therapeutic efficacy of green tea polyphenols on cellular thiols in 4-nitroquinoline 1-oxide-induced oral carcinogenesis. Chem. Biol. Interact..

[73] Srinivasan P., Sabitha K.E., Shyamaladevi C.S. (2006). Modulatory efficacy of green tea polyphenols on glycoconjugates and immunological markers in 4-nitroquinoline 1-oxide-induced oral carcinogenesis—A therapeutic approach. Chem. Biol. Interact..

[74] Wolter K.G., Wang S.J., Henson B.S., Wang S., Griffith K.A., Kumar B., Chen J., Carey T.E., Bradford C.R., D’Silva N.J. (2006). (−)-gossypol inhibits growth and promotes apoptosis of human head and neck squamous cell carcinoma *in vivo*. Neoplasia.

[75] Vidjaya Letchoumy P., Chandra Mohan K.V., Stegeman J.J., Gelboin H.V., Hara Y., Nagini S. (2008). Pretreatment with black tea polyphenols modulates xenobiotic-metabolizing enzymes in an experimental oral carcinogenesis model. Oncol. Res..

[76] Li N., Sun Z., Han C., Chen J. (1999). The chemopreventive effects of tea on human oral precancerous mucosa lesions. Proc. Soc. Exp. Biol. Med..

[77] Schwartz J.L., Baker V., Larios E., Chung F.L. (2005). Molecular and cellular effects of green tea on oral cells of smokers: A pilot study. Mol. Nutr. Food Res..

[78] Tsao A.S., Liu D., Martin J., Tang X.M., Lee J.J., El-Naggar A.K., Wistuba I., Culotta K.S., Mao L., Gillenwater A., Sagesaka Y.M., Hong W.K., Papadimitrakopoulou V. (2009). Phase II randomized, placebo-controlled trial of green tea extract in patients with high-risk oral premalignant lesions. Cancer Prev. Res. (Phila.).

[79] Han C. (2011). Studies on tea and health. Wei Sheng Yan Jiu.

[80] Halder A., Raychowdhury R., Ghosh A., De M. (2005). Black tea (Camellia sinensis) as a chemopreventive agent in oral precancerous lesions. J. Environ. Pathol. Toxicol. Oncol..

[81] Rossi M., Garavello W., Talamini R., Negri E., Bosetti C., Dal Maso L., Lagiou P., Tavani A., Polesel J., Barzan L., Ramazzotti V., Franceschi S., La Vecchia C. (2007). Flavonoids and the risk of oral and pharyngeal cancer: A case-control study from italy. Cancer Epidemiol. Biomark. Prev..

[82] Ide R., Fujino Y., Hoshiyama Y., Mizoue T., Kubo T., Pham T.M., Shirane K., Tokui N., Sakata K., Tamakoshi A., Yoshimura T. (2007). A prospective study of green tea consumption and oral cancer incidence in japan. Ann. Epidemiol..

